# Copper@polypyrrole nanocables

**DOI:** 10.1186/1556-276X-7-521

**Published:** 2012-09-25

**Authors:** Jullieth Suárez-Guevara, Omar Ayyad, Pedro Gómez-Romero

**Affiliations:** 1 Centro de Investigación en Nanociencia y Nanotecnología, CIN2 (CSIC-ICN), Campus UAB, Bellaterra, Barcelona, 08193, Spain; 2 MATGAS Research Center, Campus UAB, Bellaterra, Barcelona, 08193, Spain

**Keywords:** Copper nanocables, Nanowires, Polymer-coated metal nanocables, Conducting polymers, Metal-conducting polymer nanostructures, Hydrothermal synthesis, Hybrid materials

## Abstract

A simple hydrothermal redox reaction between microcrystalline CuOHCl and pyrrole leads to the isolation of striking nanostructures formed by polypyrrole-coated copper nanocables. These multicomponent cables that feature single-crystalline face-centered cubic Cu cores (*ca*. 300 nm wide and up to 200 μm long) are smoothly coated by conducting polypyrrole, which in addition to its functionality, offers protection against oxidation of the metal core.

## Background

Nanotubes and nanowires are two of the most striking objects being developed by nanotechnologists. Their synthesis and properties have been studied and, to a certain extent, mastered, in just the last two decades.

Many different types of materials have been fabricated in the form of nanowires from metals to oxides, other chalcogenides, and inorganic phases [1], as well as carbon or even polymeric materials [2]. However, the fabrication of multicomponent or core-shell nanowires is a more complex task. This has not prevented the pioneering attempts to build heterostructured nanowires of increasing complexity, since the promises of these materials are high. Notable examples of these efforts are the growth of coaxial (p-i-n) silicon nanowires for solar cells and nanoelectronic power systems, [3,4] the formation of atomic metal nanowires inside carbon nanotubes including Mo [5,6] or Cu [7,8], or the electrochemical growth of metals inside TiO_2_ nanotubes [9]. In all of these cases, very valuable complex nanomaterials were produced which were nevertheless very elaborate to fabricate.

Along the path to complex materials, a double challenge remains: to prepare more and more complex materials and to do it through simpler and simpler methods. In great contrast with costly physical methods like chemical vapor deposition or molecular beam epitaxy, the bottom-up approach typically associated to the chemical methods provides many opportunities for the development of high-throughput synthesis of nanostructured materials. But as the complexity of the target materials increase, chemical methods tend to be limited by entropy. Fortunately, specific molecular interactions come to the rescue in the form of what we call self-organization or self-assembly.

In our group we have explored the development of complex hybrid materials through this type of approach [10]. Specifically, we have recently carried out synthetic work of hybrids made of silver and biopolymers through matrix chemistry [11], as well as silver and conducting polymers through simple hydrothermal reactions [12,13]. In this way we have recently reported the isolation of ordered Ag@PPy structures we dubbed as ‘nanosnakes’ [12]. Silver or gold are noble metals that are very stable in their metallic form. On the other hand, cheaper but more reactive copper nanostructures are prone to oxidation in air/aqueous solutions. Protection of copper nanoparticles or nanostructures with polymers is an obvious approach to their stabilization. Furthermore, the use of multifunctional conducting polymers for this task could lead to novel materials with built-in multifunctionality. However, attempts to build this type of hybrid materials have been limited to the previous synthesis of Cu nanoparticles followed by coating with PPy which is prepared subsequently and limited to the coating of Cu nanoparticles [14,15].

We report here the first synthesis of polypyrrole-coated copper nanowires (Cu@PPy), which, in great contrast with the mentioned silver nanosnakes, are formed not by attached nanoplates but by truly nanometric rods of metallic copper coated with polypyrrole forming wires of a few hundred nanometers in diameter and lengths up to a few hundred microns. Furthermore, these coated nanocables are prepared in a single reaction step where the simultaneous oxidation of pyrrole and reduction of copper precursor takes place.

## Methods

### Materials

Pyrrole (Sigma-Aldrich Corporation, St. Louis, MO, USA) was vacuum-distilled prior to its use. Greenish CuOHCl (J. T. Baker Chemical Company Phillipsburg, NJ, USA) was confirmed through powder X-ray diffraction and was used as received.

### Synthesis

In a typical synthesis, 20 mL of deionized water and 0.2 g of CuOHCl were placed into a 25 mL screw-capped Pyrex bottle. The resulting suspension was stirred for 10 min. Then 0.3 g of pyrrole was added and the mixture was stirred for 10 more minutes. The bottle was then tightly closed with a Teflon screw-cap and heated at 150°C for different times. After reaction the resulting black suspensions were filtered through 0.8 μ cellulose acetate filters; the black solid was washed with deionized water and dried at 50°C.

### Characterization

Transmission electron microscopy (TEM) images and selected area electron diffraction (SAED) patterns were obtained on a JEM-1210 by JEOL Ltd., Tokyo, Japan at 120 kV. Scanning electron microscope (SEM) images were taken with a Quanta 200 environmental scanning electron microscope using field-emission gun by FEI (FEI Company, Hillsboro, OR, USA). X-ray diffraction (XRD) analyses were performed with a Siemens D5000 diffractometer (Siemens AG, Munich, Germany) (λ = 1.54056). The FTIR spectra of all the samples were measured with a PerkinElmer model Spectrum One spectrometer (PerkinElmer Inc., Waltham, MA, USA) connecting with attenuated total reflectance accessory. UV-visible spectra were recorded using Cary 5 (Varian Medical Systems Inc., Palo Alto, CA, USA) UV–vis-NIR high-resolution optical spectrophotometer.

## Results and discussion

The key conditions for getting these extraordinary self-assembled nanostructures include the use of hydrothermal conditions (under equilibrium at high temperatures) plus carrying out heterogeneous reactions between an oxidizing solid and a monomer solution prone to oxidative polymerization. Thus, in the present case we used solid Cu(OH)Cl to oxidize pyrrole in an aqueous solution at 150°C under hydrothermal autogenous pressure. The synthesis procedure is described in detail in the experimental section.

**Figure 1:**
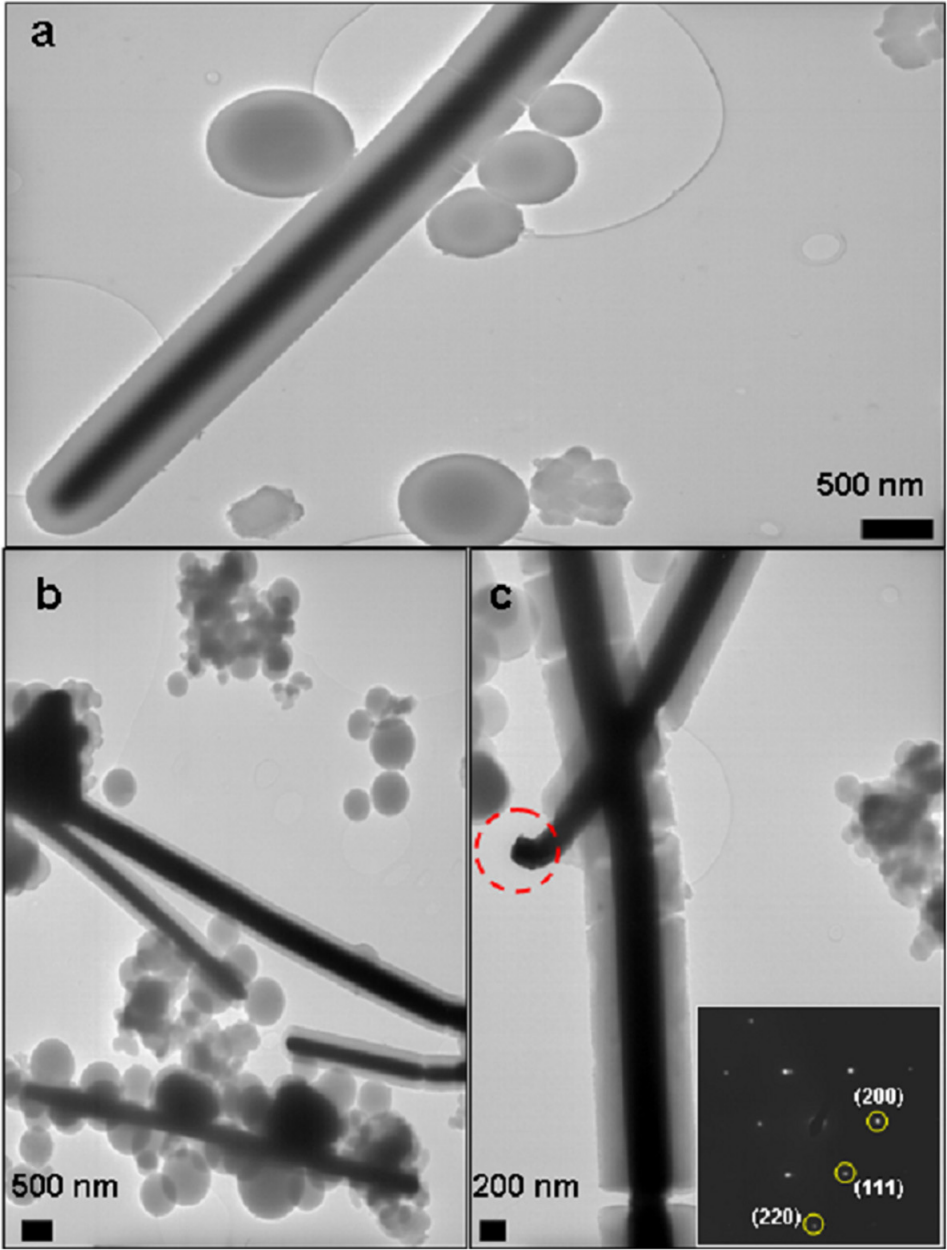
**TEM image of Cu@PPy nanocables prepared by hydrothermal synthesis.** (**a**-**c**) show different regions of the sample Cu@PPy nanocables that were prepared by hydrothermal synthesis of Py:CuOHCl, with molar ratio of 2:0.78 and period of 72 h at 150°C. Inset in (**c**) shows the SAED of the region marked.

**Figure 2:**
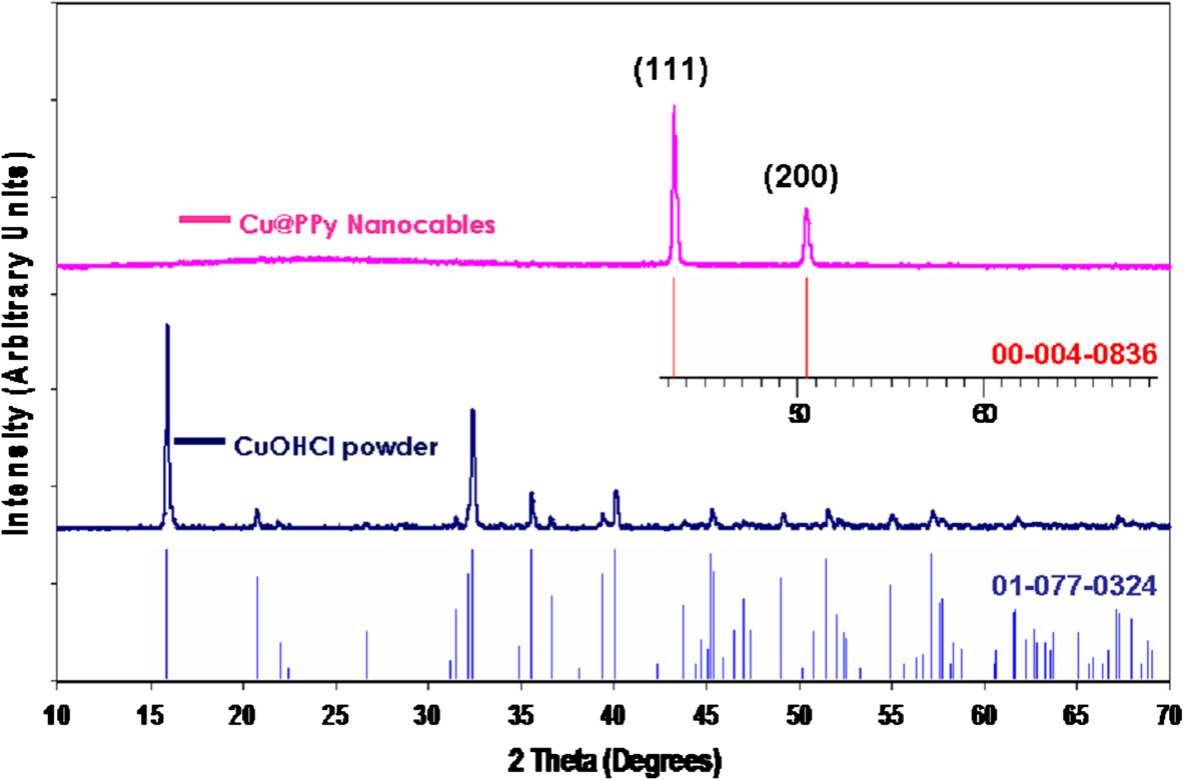
**X-ray diffraction patterns of the synthesized polypyrrole coated copper nanocables and copper chloride hydroxide powder.** This figure shows the XRD patterns of the synthesized polypyrrole coated copper nanocables (top) and the copper chloride hydroxide powder (middle dark blue line). The upper red vertical ticks mark the Bragg positions for fcc single crystal copper (ICDD PDF No. 040–0836). The lower light blue vertical ticks show the Bragg peaks of the monoclinic single crystal CuOHCl precursor (ICDD PDF No. 077–0324).

Figure 1 shows the representative TEM images of samples obtained as detailed in the experimental procedure (pyrrole 0.2960 g and CuOHCl 0.1995 g) at 150°C for various periods of time. These samples contain a large number of nanocables structures together with abundant globular formations. The high contrast of the nanocable cores suggested their metallic nature, which was confirmed by SAED of an isolated wire. This, together with powder X-ray diffraction of the bulk materials (Figure 2) ruled out the possibility that the cores could be made of CuOHCl or copper oxides and confirmed the growth of face-centered cubic (fcc) copper. Two diffraction peaks of copper nanocables (Figure 2) at 2*θ* = 43.26°, 50.46° could be observed clearly from the XRD pattern, which could be indexed as (111) and (200) planes of fcc copper. It should also be remarked that the metallic copper core is single-crystalline in nature as showed by the SAED pattern. The SAED patterns of the marked region from Figure 1c are shown in bottom inset of that figure. The *d*-spacing calculation was carried out using Equation 1.1, where *d* is the *d*-spacing, λ is the wavelength of the electron beam, *L* is the camera length (in millimeters); the constant (λ*L*) is the camera constant which equals to 22.5 Å mm; and *R* is radius of the diffraction pattern (in millimeters).

(1) $$d=\frac{\lambda L}{R}$$

Table 1 shows the *d*-spacings calculated for the SAED pattern as shown in Figure 1c (inset). It can be seen that the *d*-spacings for copper fits well with the experimental data, which also agree with the XRD of Cu@PPy (Figure 2).

**Table 1:** **The*d*-spacings for the experimental data (calculated from SAED), copper (ICDD PDF No. 004–0836)**

**Experimental**	**Copper**	
***d*-spacing (Å)**	**Plane (hkl)**	***d*-spacing (Å)**
2.14	<1 1 1>	2.088
1.87	<2 0 0>	1.808
1.25	<2 2 0>	1.278

According to the calculation of electron diffraction spots in Figure 1c (inset), it was revealed that the crystal planes spacing of the inner core region were 0.21 nm, 0.187, and 0.125 which corresponded to the crystal plane distances of the main diffraction peaks of copper; the crystal planes are (111), (200), and (220), respectively.

**Figure 3:**
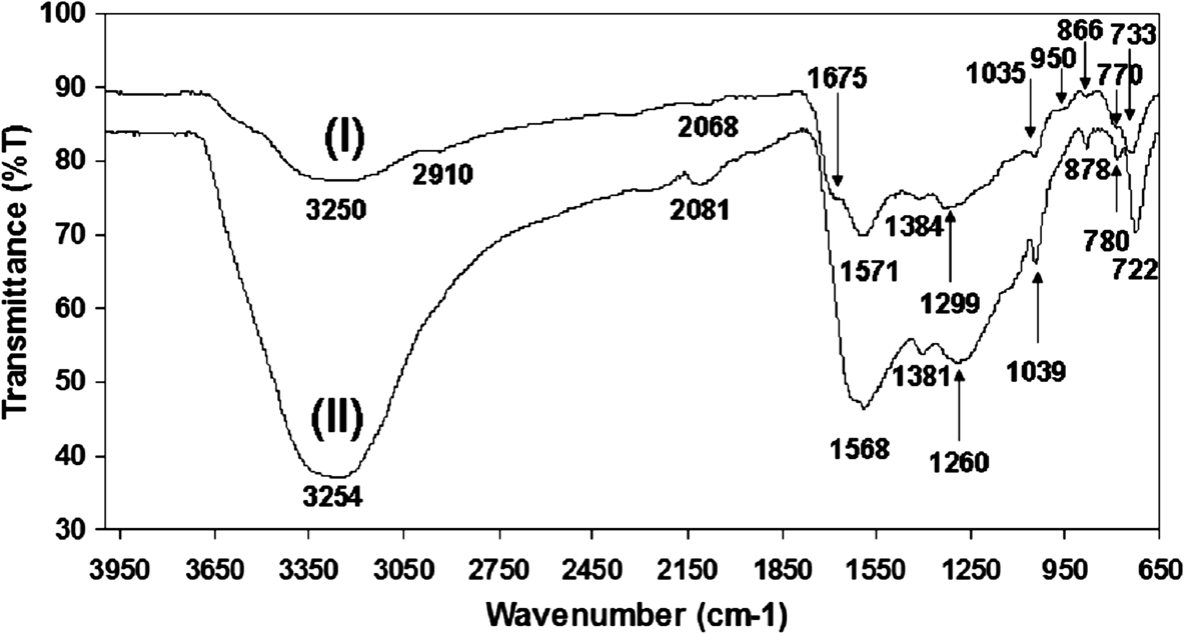
FTIR-ATR spectra of Cu@PPy sample (I) and pure pyrrole sample (II).

The Fourier transform infrared spectroscopy (FTIR) spectrum of the sample features the peaks characteristic of polypyrrole (Figure 3). (peaks at 750–780 cm^−1^ and peak near 800 cm^−1^ assigned to N-H out of plane bending absorption). The band observed near 950 cm^−1^ is due to the C-H out of plane bending. The peaks near 1,035 cm^−1^ are due to the C = C stretching of aromatic compounds. The peak near 1,384 cm^−1^ corresponds to C-N stretching and C-C vibration. The prominent bands of polypyrrole structure are the aromatic ring vibrations at 1,571 cm^−1^, C-N in plane deformation at 1,299 cm^−1^ and C-H in plane vibrations at 1,035 and 950 at 770 cm^−1^[16]. The C = O band is observed at 1,675 cm^−1^ which can be assigned to the oxidized pyrrole rings in Cu@PPy. The broad band at 3,300 cm^−1^ is assigned to O-H stretching vibration. The frequency at 2,910 cm^−1^ refers to the stretching vibration of the C-H bond [17]. Thus, we conclude that pyrrole was oxidized *in situ* by CuOHCl, leading to the nanostructures formed by the reaction products, i.e., reduced metallic copper (single-crystalline) and oxidized polypyrrole. The remarkable finding is how this reaction under hydrothermal conditions leads to such a complex and well-ordered nanostructure through a simple synthesis. 

**Figure 4:**
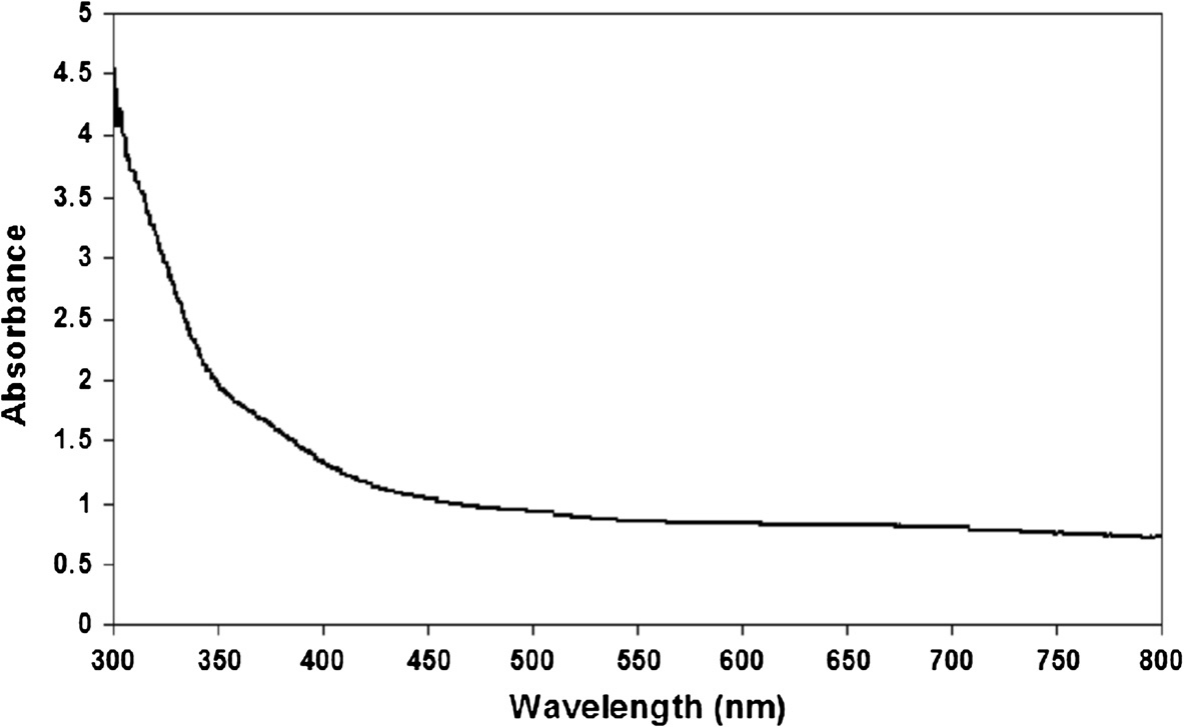
The UV–vis absorption spectra of the synthesized Cu@PPy liquid phase.

The ultraviolet (UV)-vis spectra of the synthesized polypyrrole-coated copper nanocables at liquid phase (pH = 6.4) (Figure 4) show no absorption peaks corresponding to copper particles, which had a characteristic absorption peak at 580 nm [18]. These results agree well with the experimental results obtained, since copper is always detected coated with polypyrrole. The Cu@PPy composites are normally settled down in the reaction vessel. Noteworthy, the UV–vis shows no peaks that could indicate the presence of Cu^2+^-pyrrole complex, which in turn indicates that all the Cu^2+^ ions have reacted with pyrrole at the end of the preparation process. 

It should be noted that PPy coats these nanocables very smoothly, but also, that excess PPy forms the conspicuous globular formations. The excess PPy in the form of nanospheres is consistent with the initial composition of the mixture 2:0.78 Py:Cu. We tried to adjust this ratio to optimize the volume fraction of nanocables but found that a 1:1 Py:Cu ratio would not lead to the formation of nanocables. The optimization of synthetic procedures and the isolation of pure nanocables will be the subject of future work.

**Figure 5:**
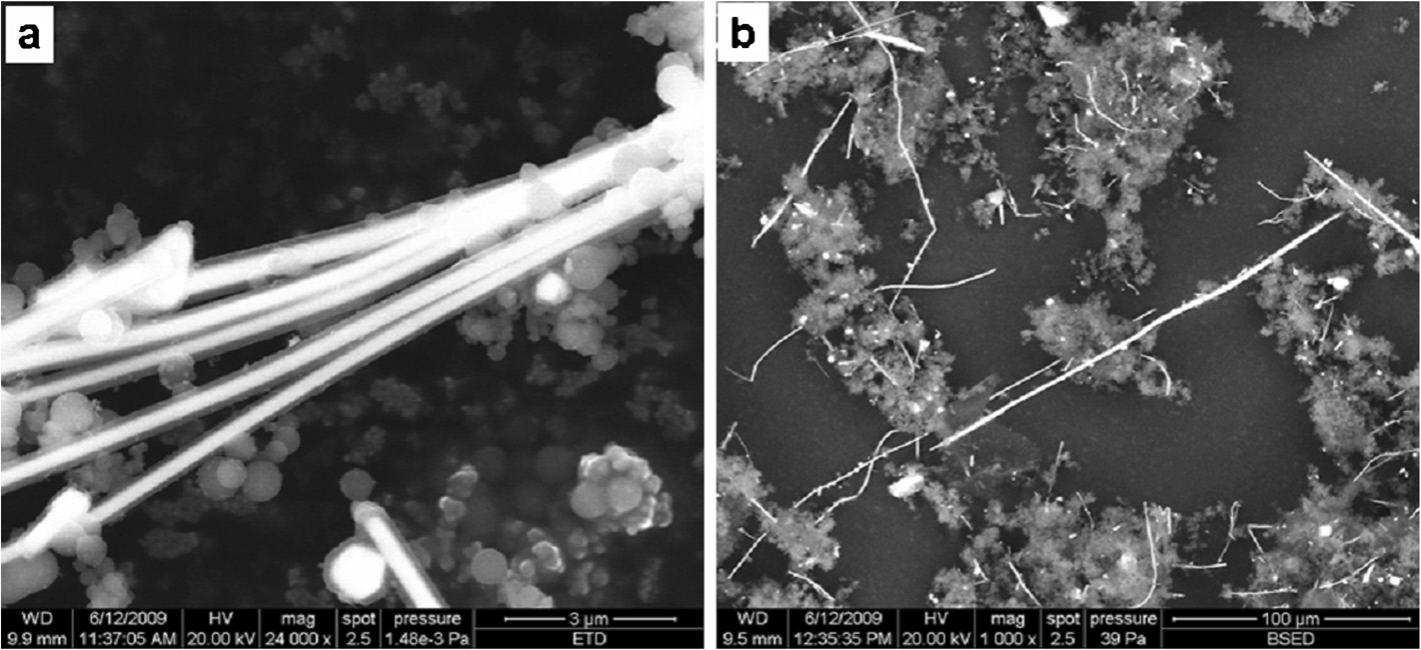
**SEM image of a sample obtained by hydrothermal synthesis of Py:CuOHCl. a**: image of a sample obtained by hydrothermal synthesis of Py:CuOHCl with molar ratio of 2:0.78 and period of 72 h at 150°C, **b**: Lower magnification view of the sample.

Concerning the size and shape of the Cu@PPy nanocables, Figure 1 shows a few of them featuring Cu cores of 290 ± 45 nm wide covered by shells of PPy approximately 130 nm wide. The length of these nanocables is difficult to asset in a statistically meaningful way. However, we have detected with TEM single nanocables as long as 85 μm (600 nm wide) and SEM images show even larger nanocables (Figure 5).

## Conclusions

The reaction of Copper(I) hydroxochloride with pyrrole under hydrothermal conditions leads to the reduction of the former to metallic Cu and the oxidative polymerization of pyrrole. Interestingly, under the conditions used, both solids grow together forming long PPy-coated nanocables, i.e., Cu@PPy nanocables featuring single-crystal fcc Cu cores. The fascinating possibilities for further work on these nanostructures are multiple. They could be used, for instance, as precursors for the fabrication of carbon-coated copper nanowires, which have been targeted as useful materials for a variety of applications [7]. But in addition to that possibility, these nanocables would deserve their own attention. Since they are formed by a metallic-conducting core and a p-doped conducting polymer coating, the first intriguing question arises as to what special conductivity and electron-transfer properties these nanocables could have. Their quantum-size effects, the nanoelectrochemical and sensing performance of the materials, as well as the prospective research on synthesis of other related nanostructures by the same simple method, all will deserve and surely get due attention.

## Competing interests

The authors declare that they have no competing interests.

## Authors' contributions

JSG carried out the synthesis and basic characterization of the materials, OA contributed to both synthesis and characterization and helped in the writing and publication process. PGR conceived and directed the study and wrote the manuscript. All authors read and approved the final manuscript.

## Authors' information

JSG is a Ph. D. student working under the supervision of PGR. OA is a post-doctorate researcher in the NEO-Energy group. He received his Ph. D. in Materials Science from Barcelona University last 2011. Currently, he is working on cathode nanomaterials for energy conversion and storage, nanoscale coating, and safety enhancement of the Li-ion batteries. PGR is a full research professor and group leader of the NEO-Energy group. He is also the vice-director of MATGAS Research Center and presently leading its research projects on energy-related materials and devices, Li batteries, supercapacitors, fuel cells, and solar energy.
